# Validation of a survival benefit estimator tool in a cohort of European kidney transplant recipients

**DOI:** 10.1038/s41598-020-74295-3

**Published:** 2020-10-13

**Authors:** Armando Coca, Carlos Arias-Cabrales, Ana Lucía Valencia, Carla Burballa, Juan Bustamante-Munguira, Dolores Redondo-Pachón, Isabel Acosta-Ochoa, Marta Crespo, Jesús Bustamante, Alicia Mendiluce, Julio Pascual, María José Pérez-Saéz

**Affiliations:** 1 Department of Nephrology, Hospital Clínico Universitario de Valladolid, Avda. Ramón y Cajal SN, 47003 Valladolid, Spain; 2 Nephrology Department, Hospital del Mar, Barcelona, Spain; 3 Nephrology Department, Hospital Universitario Río Hortega, Valladolid, Spain; 4 Cardiac Surgery Department, Hospital Clínico Universitario, Valladolid, Spain; 5 Medicine, Dermatology and Toxicology Department, School of Medicine, Universidad de Valladolid, Valladolid, Spain; 6 Grupo de Trabajo de Jóvenes Nefrólogos de la Sociedad Española de Nefrología (JovSEN), Madrid, Spain

**Keywords:** Prognosis, Nephrology, Renal replacement therapy

## Abstract

Pre-transplant prognostic scores help to optimize donor/recipient allocation and to minimize organ discard rates. Since most of these scores come from the US, direct application in non-US populations is not advisable. The Survival Benefit Estimator (SBE), built upon the Estimated Post-Transplant Survival (EPTS) and the Kidney Donor Profile Index (KDPI), has not been externally validated. We aimed to examine SBE in a cohort of Spanish kidney transplant recipients. We designed a retrospective cohort-based study of deceased-donor kidney transplants carried out in two different Spanish hospitals. Unadjusted and adjusted Cox models were applied for patient survival. Predictive models were compared using Harrell’s C statistics. SBE, EPTS and KDPI were independently associated with patient survival (p ≤ 0.01 in all models). Model discrimination measured with Harrell’s C statistics ranged from 0.57 (KDPI) to 0.69 (SBE) and 0.71 (EPTS). After adjustment, SBE presented similar calibration and discrimination power to that of EPTS. SBE tended to underestimate actual survival, mainly among high EPTS recipients/high KDPI donors. SBE performed acceptably well at discriminating post-transplant survival in a cohort of Spanish deceased-donor kidney transplant recipients, although its use as the main allocation guide, especially for high KDPI donors or high EPTS recipients requires further testing.

## Introduction

The survival benefit of kidney transplantation (KT) compared with remaining on dialysis and the rising prevalence of chronic kidney disease result in a continuous increase in the allograft demand/supply ratio.

As a consequence of this, changes in the acceptance criteria of donors into transplant programs have been implemented in order to increase the donor pool^1–5^. However, the improvement in survival associated with transplantation in old recipients (≥ 65 years) who receive marginal kidneys remains unclear^6,7^.

In 2014, the US Organ Procurement and Transplantation Network (OPTN) started a new kidney allocation system (KAS) based in donor (Kidney Donor Profile Index—KDPI) and recipient (Estimated Post Transplant Survival—EPTS) scores. Their aims were to improve donor/recipient matching and to decrease organ discard rates^8^. KDPI is a percentile measure calculated with ten donor variables which is currently applied in all deceased US donors to estimate the risk of post-transplant graft failure^9,10^. EPTS is also a percentile measure that includes four recipient variables and provides information about potential recipient survival after receiving a KT. Briefly, higher EPTS or KDPI scores indicate lower quality organs and worse expected survival of recipients. The OPTN KAS allocates kidneys with lower KDPI scores to candidates with lower EPTS scores to obtain the highest benefit^11^. Recently, a new scoring tool (Survival Benefit Estimator, SBE) has been developed to estimate post-transplant survival benefit based on KDPI and EPTS. SBE calculates the probability of 5-year post-transplant survival after receiving a kidney allograft from a specific donor. Results suggest that receiving a KT may be associated with lower mortality risk when compared to remaining on the waitlist across any possible KDPI and EPTS combination^12^.

Nevertheless, application of these scores in non-US patients might not be advisable due to the intrinsic differences existing between transplant programs in the US and other countries such as Spain^4,13,14^. In 2017, 45.7% of Spanish donors were ≥ 60 years, while only 4.9% of US donors were 65 years or older^15^. There were also significant differences regarding donor cause of death, diabetes or hypertension prevalence^15,16^. However, recent studies have shown the potential benefit that the application of these scores might have in non-US populations. KDPI has shown to improve the expanded criteria donor classification in order to provide an estimation of graft survival in a cohort of Spanish KT recipients^17^ while the introduction of the Kidney Donor Risk Index in the decision-making process to accept or decline deceased donor kidneys in a Eurotransplant center helped to increase its transplantation rate by 26%^17–20^.

The main objective of this study was to assess the prognostic performance of SBE in a two-center Spanish kidney transplant cohort. Secondary objectives of the study were (1) to evaluate the effectiveness of EPTS and KDPI when predicting post-transplant survival, (2) to describe the distribution of donors and recipients in our sample according to KDPI and EPTS scores and (3) to compare the characteristics of the study subjects with the US dataset.

## Patients and methods

### Study design and data collection

We designed a retrospective cohort study with adult (age ≥ 18 years) deceased-donor KT recipients performed from January 2000 to July 2015 in two different hospitals in Spain. KT from donors younger than 18 years old, living donors, multiple-organ transplants and recipients who were lost to follow-up during the first 5 years after transplantation were excluded from the analysis. Anthropometric, clinical, and analytical variables were collected from each site’s local transplant database. Time on dialysis was calculated considering all dialysis periods for patients that received more than one graft.

This project was conducted in accordance with the ethical standards of the 2000 Declaration of Helsinki as well as the 2008 Declaration of Istambul. No organs were procured from prisoners. The Institutional Review Boards and Ethics committees of both centers approved the study (PI 18-1051, Comité de Ética de la Investigación con Medicamentos, Área de Salud Valladolid Este). Informed consent was waived by the same authority that approved the study (Comité de Ética de la Investigación con Medicamentos, Área de Salud Valladolid Este) due to the observational and retrospective nature of the study. The study is reported in accordance with guidelines set forth in the 2007 STROBE statement^21^.

### Survival estimation and actual survival

Predicted 5-year patient survival was calculated using the prognostic score developed by Bae et al.^12^, available at https://www.transplantmodels.com/kdpi-epts/. Briefly, SBE estimates the survival benefit (i.e. the absolute reduction in mortality risk expressed in percentage points) for a distinct recipient of receiving a kidney from a specific donor using a combination of EPTS and KDPI scores.

Actual post-transplant patient survival was defined as the time from KT to death, censoring by the end of the study. All patients were followed-up long enough to establish 60-month survival, regardless the allograft status (functioning or not).

The following donor variables were used to calculate KDPI: Age, race, height, weight, hypertension, diabetes, serum creatinine, hepatitis C seropositivity and cause of death, using the methods depicted in the OPTN site. KDPI scores range from 0 to 100% and indicate the potential longevity and quality of a kidney graft compared to a reference population^9^. EPTS was calculated using recipient variables including age, diabetes, time on dialysis and previous solid organ transplant, following the methods described in the OPTN site. EPTS scores vary from 0 to 100%. Patients with lower EPTS scores should experience more years of graft function from high-longevity kidneys compared to subjects with higher EPTS scores^11^.

To examine the association of SBE score with patient survival, we stratified our sample by SBE quintiles. Adjusted models were built using SBE, EPTS or KDPI and other recipient variables of clinical relevance (sex, hypertension, ischemic heart disease, congestive heart failure, peripheral vascular disease, stroke and hepatitis C status). Those variables already considered in the calculation of KDPI or EPTS scores were not included in this last analysis.

### Statistical analysis

Data are expressed as mean and standard deviation (SD) or median and range for non-normal distributions. Qualitative variables are described as frequencies and percentages. T tests and analyses of variance were used to assess continuous variables. Chi-square test was used to compare categorical data between groups.

To address missing values, we first applied Little’s missing completely at random test to assess the assumption of missing completely at random for multivariate quantitative data^22^, which was non-significant (P > 0.05). Multiple imputation by fully conditional specification was applied to the dataset. A total run length of 1000 iterations was used, creating 10 imputed data sets to take into account uncertainty associated with missing data. All variables needed to calculate EPTS and KDPI score were included in the procedure (donor age, race, height, weight, hypertension, diabetes, serum creatinine, hepatitis C seropositivity and cause of death; recipient age, diabetes, time on dialysis and previous solid organ transplant). Final results were averaged across the created sets.

To compare the original SBE cohort with our study cohort, mean and standard deviation were estimated on the basis of the reported median and range according to the method developed by Hozo SP et al.^23^. Quintile distribution according to SBE score (Q1: 0–75; Q2: 75.1–83.5; Q3: 83.6–88.8; Q4: 88.9–93; Q5: 93.1–100) was used to obtain groups of comparable size. Overall survival probabilities for each group were estimated using the Kaplan–Meier method and compared by the log-rank test. Hazard ratios (HRs) with 95% confidence interval (CI) were calculated using unadjusted and adjusted Cox proportional hazards regression modelling. To ensure confidence interval coverage and type I error rate as defined by Vitinghoff et al.^24^ the study sample included at least ten events per predictor variable. Model discrimination was estimated using Harrell’s C statistic^25^. Harrell’s C estimates the proportion of correct predictions, serving as a concordance index. The results of Harrell’s C index varied from 0.5 (no discrimination) to 1 (perfect discrimination). The Benjamini–Hochberg procedure was used to control for the false discovery rate^26^. We examined the Akaike information criterion (AIC), a technique based on in-sample fit to estimate the likelihood of a model to predict future values. The statistical significance level was set at p < 0.05. Statistical analysis was performed using IBM SPSS v.22 (IBM SPSS, Chicago, IL, USA), GraphPad Prism v.7 (GraphPad Software, San Diego, CA, USA) and Microsoft Excel (Microsoft, Redmond, WA, USA).

## Results

### Baseline characteristics

**Figure 1:**
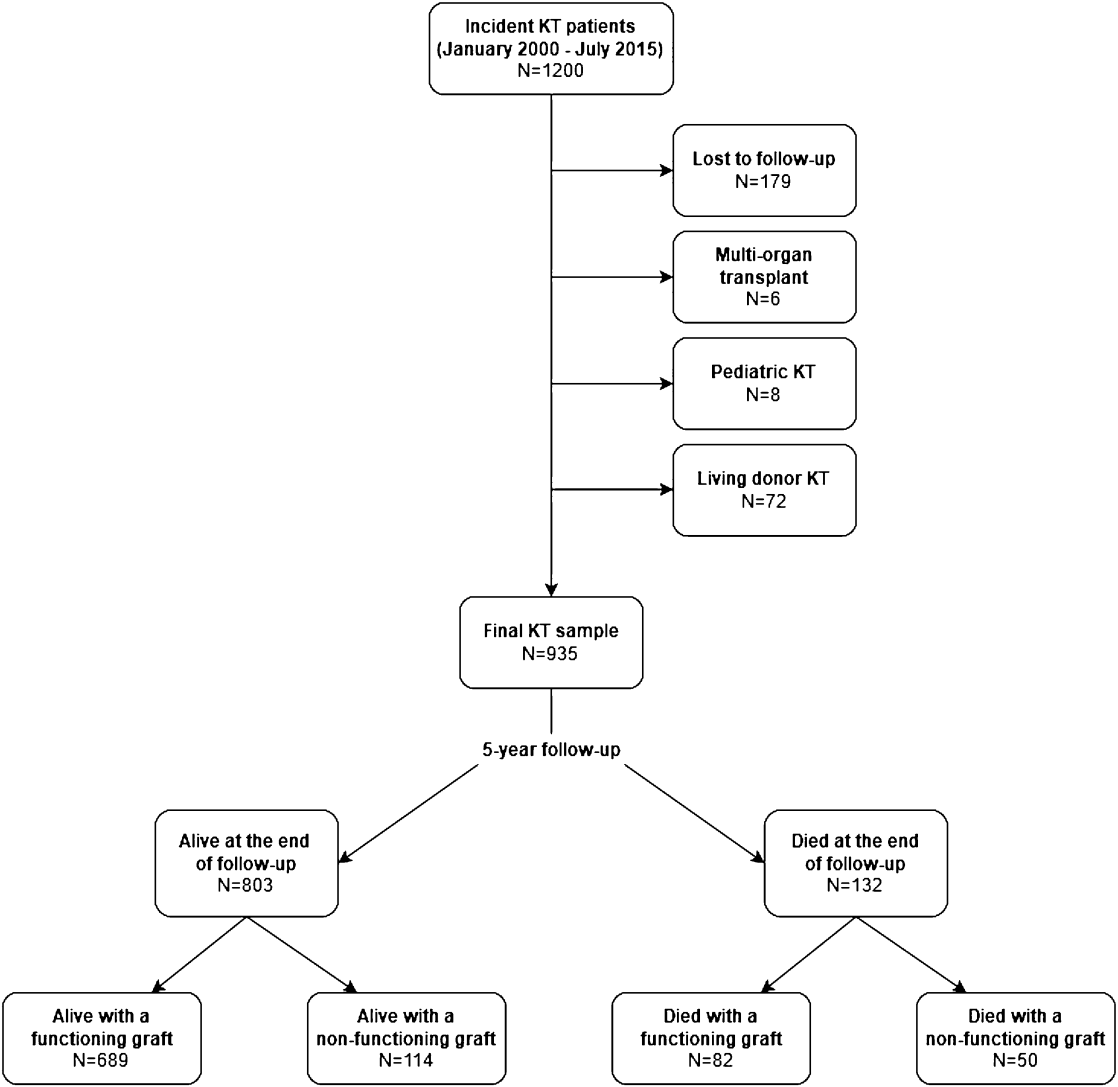
Flow chart of patients included in the study.

A total of 1200 KT were performed in two Spanish hospitals between January 2000 and July 2015. The final analysis included 935 KT patients. Flow-chart of patients is represented in Fig. 1.

Regarding baseline characteristics, a comparative between Bae´s cohort and our study cohort is summarized in Table 1.

**Table 1:** Comparison of Bae’s and Spanish cohorts.

	Bae's cohort N = 120,818	Spanish cohort N = 935	P value
**Recipients**			
Age, years (median, IQR)	54 (44–63)	58 (48–67)	< 0.001
Sex, male %	60.3	63.6	< 0.001
**Race, %**			< 0.001
White	43	92.2	
Black	33.1	2	
Hispanic	15.6	4.1	
Other	8.4	1.7	
**Primary renal disease, %**			–
Polycystic kidney disease	-	14.4	
Glomerulonephritis	-	19.8	
Tubulo-interstitial disease	-	11.6	
Diabetic nephropathy	-	16.9	
Vascular disease + /− HTN	-	8.5	
Others	-	7.5	
Unknown	-	21.3	
HTN, %	87.9	90.7	< 0.001
Diabetes, %	35	8.8	< 0.001
Ischemic heart disease, %	-	9.1	-
Congestive heart failure, %	-	27	-
Peripheral vascular disease,%	-	27.4	-
Stroke, %	-	8.9	-
Hepatitis C, %	-	3	-
**Peak PRA, %**			< 0.001
0–9	56.1	95.9	
10–79	25.7	3.6	
80–100	18.1	0.4	
Re-transplantation, %	14.7	11.3	< 0.001
Dialysis vintage, years median (IQR)	3.4 (1.6–5.6)	1 (0–3)	< 0.001
EPTS median (IQR)	45 (20–74)	36 (18–63)	< 0.001
**Donors**			
Age, years median (IQR)	40 (25–51)	56 (46–67)	< 0.001
Sex, male %	60.2	56.5	< 0.001
**Race, %**			< 0.001
White	68.8	97.1	
Black	14.1	1.2	
Hispanic	13.7	1.1	
Other	3.3	1.1	
Weight, kg median (IQR)	78.9 (66–93)	75 (65–85)	< 0.001
Height, cm median (IQR)	171 (163–179)	168 (160–175)	< 0.001
HTN, %	28	34.8	< 0.001
Diabetes, %	7.3	10.6	< 0.001
Serum creatinine, mg/dl median (IQR)	0.9 (0.7–1.3)	0.81 (0.61–1)	< 0.001
Donation after cardiac death, %	15.2	12.3	< 0.001
Hepatitis C, %	2.6	1.5	< 0.001
KDPI	49 (25–71)	70 (45–89)	< 0.001
Cold ischemic time. hours median (IQR)	16.9 (11.5–23)	15 (12–18)	< 0.001

*EPTS* estimated post transplant survival score, *HTN* hypertension, *IQR* interquartile range, *KDPI* kidney donor profile index, *PRA* panel reactive antibodies.

Spanish recipients and donors were older and predominantly Caucasians (92.2%). Spanish recipients showed lower prevalence of diabetes, less time on dialysis before KT and lower rates of re-transplantation. The proportion of pre-sensitized patients was higher in Bae´s cohort, while KDPI scores were higher and EPTS scores were lower in the Spanish cohort.

The study sample was divided into five groups according to SBE value distribution. Table 2 summarizes recipient, donor and transplant related data.

**Table 2:** Comparison of recipients, donors and transplantation characteristics according to SBE quintile distribution.

	SBE estimated post-transplant survival (quintiles)					P value
	1st Q (worst)	2nd Q	3rd Q	4th Q	5th Q (best)	
N	188	187	189	191	180	
Age, years median (IQR)	72 (68–75)	65 (60–69)	58 (52–63)	52 (46–56)	40 (33–46)	< 0.001
Male sex, %	64.4	65.2	62.4	59.7	66.5	0.684
**Race, %**						
White	98.9	97.9	89.4	90.1	84.4	< 0.001
Black	0	0	3.2	2.1	5	0.002
Hispanic	1.1	1.6	5.8	4.7	7.3	0.04
Other	0	0.5	1.6	3.1	3.4	0.009
**Primary renal dis. %**						
Polycystic kidney dis	11.2	9.6	17.5	15.8	17.8	0.077
Glomerulonephritis	12.8	24.6	20.1	18.9	22.8	0.046
Tubulo-interstitial dis	11.7	10.7	12.2	12.1	11.1	0.991
Diabetic nephropathy	21.8	22.5	13.2	17.9	8.9	0.002
Vascular dis. + /− HTN	10.1	9.1	9	7.4	7.1	0.832
Others	9.6	5.9	6.3	5.8	10	0.32
Unknown	22.8	17.6	21.7	22.1	22.3	0.73
HTN, %	91	90.4	88.8	93.2	90	0.685
Diabetes, %	27.7	9.1	4.2	2.6	0	< 0.001
Ischemic heart dis, %	14.9	14.4	7.9	6.3	1.7	< 0.001
Congestive heart failure, %	25	31	30.7	27.2	20.6	0.135
Peripheral vascular dis, %	33.5	41.2	28	21.5	12.3	< 0.001
Stroke/TIA, %	11.7	9.7	12.8	6.3	3.9	0.014
Hepatitis C, %	5.9	0.5	2.1	3.7	2.8	0.041
**Peak PRA, %**						
0–9	93.6	97.3	94.7	96.3	97.8	0.207
10–79	6.4	1.6	4.2	3.7	2.2	0.113
80–100	0	1.1	1.1	0	0	0.201
Previous transplants, %	14.4	15.5	11.1	8.4	7.2	0.047
DV, years median (IQR)	2 (1–4)	1 (0–3)	1 (0–2)	1 (0–2)	1 (0–2)	< 0.001
EPTS, median (IQR)	78 (68–88)	55 (44–69)	34 (25–46)	22 (16–31)	9 (5–14)	< 0.001
**Donor factors**						
Age. years median (IQR)	70 (62–76)	62 (50–66)	59 (51–66)	53 (45–58)	44 (34–50)	< 0.001
Male sex, %	64.4	65.2	62.4	59.7	66.5	0.684
**Race, %**						
White	98.9	97.9	89.4	90.1	84.4	< 0.001
Black	0	0	3.2	2.1	5	0.002
Hispanic	1.1	1.6	5.8	4.7	7.3	0.009
Other	0	0.5	1.6	3.1	3.4	0.041
Weight, kg median (IQR)	75 (67–82)	75 (65–82)	73 (65–80)	75 (65–85)	75 (65–85)	0.170
Height, cm median (IQR)	165 (160–172)	165 (160–170)	170 (160–174)	170 (163–175)	170 (163–180)	< 0.001
HTN, %	59	43.9	25.9	30.4	13.9	< 0.001
Diabetes, %	19.1	17.1	7.9	7.9	0.6	< 0.001
SCr, mg/dl median (IQR)	0.8 (0.6–1)	0.82 (0.65–1)	0.8 (0.6–1)	0.82 (0.6–1)	0.89 (0.65–1)	0.342
DCD, %	6.4	11.8	12.7	14.7	16.1	0.048
Hepatitis C, %	2.7	2.1	1.6	1	0	0.262
KDPI, median (IQR)	94 (84–97)	80 (60–92)	74 (54–86)	58 (40–75)	38 (24–50)	< 0.001
CIT, hours median (IQR)	15 (12–19)	15 (12–18)	15 (12–19)	15 (12–18)	15 (12–18)	0.945

*CIT* cold ischemia time, *DCD* donation after cardiac death, *Dis* disease, *DV* dialysis vintage, *EPTS* estimated post transplant survival score, *HTN* hypertension, *IQR* interquartile range, *KDPI* kidney donor profile index, *PRA* panel reactive antibodies, *Q* quintile, *SBE* survival benefit estimator score, *SCr* serum creatinine, *TIA* transient ischemic attack.

Patients with worse estimated post-transplant survival were older, had longer dialysis vintage and suffered more frequently from diabetes or cardiovascular disease. These patients received kidneys from older donors with a higher prevalence of hypertension and diabetes. There was a higher percentage of KT from cardiac-death donors among recipients with better estimated post-transplant survival.

### Distribution of KDPI and EPTS scores in the Spanish cohort

**Figure 2:**
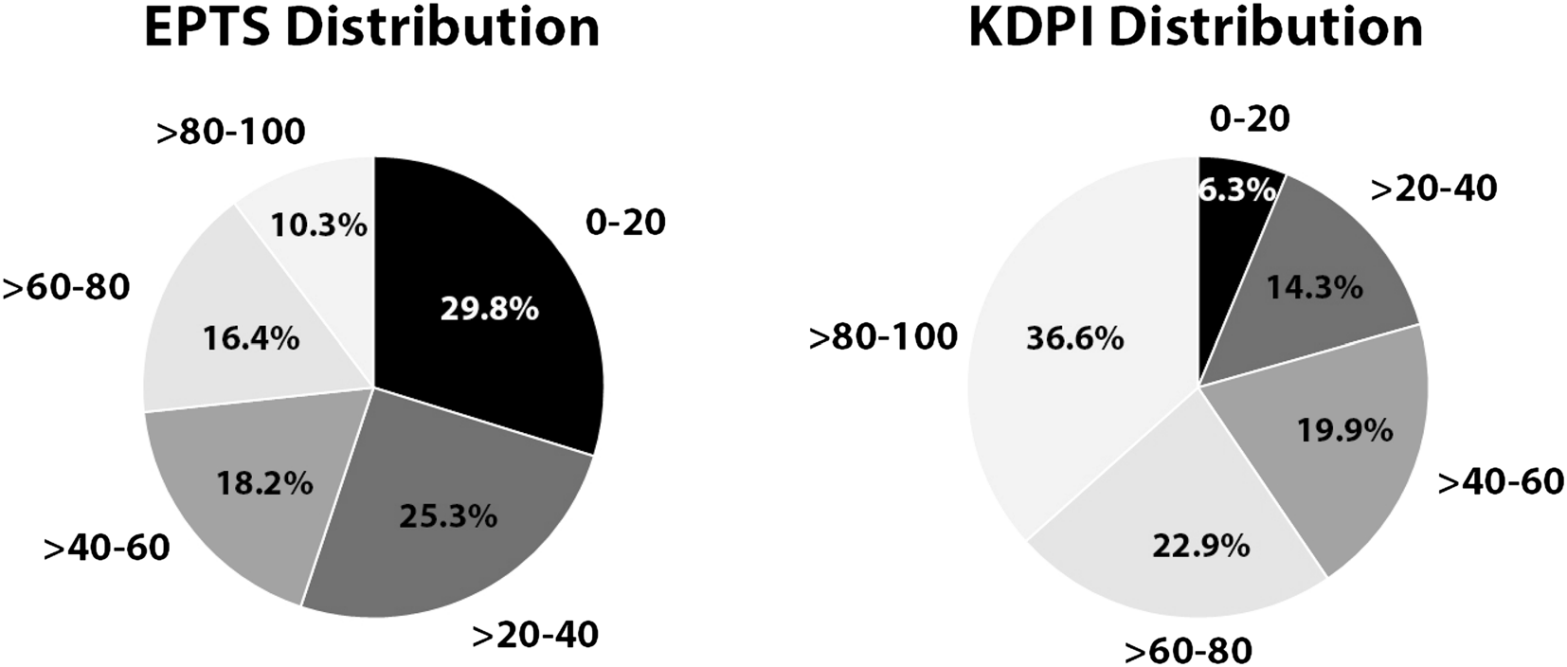
Distribution of donors and recipients according to KDPI and EPTS scores.

In the Spanish cohort 36.6% of donors presented with KDPI > 80%, while a low number of recipients (10.3%) had high EPTS scores (EPTS > 80). The majority of recipients with EPTS > 85 (62.9%) received a kidney from a KDPI > 85 donor. The distribution of KDPI and EPTS scores in our cohort is shown in Fig. 2.

### Comparison of 5-year predicted vs actual survival

**Figure 3:**
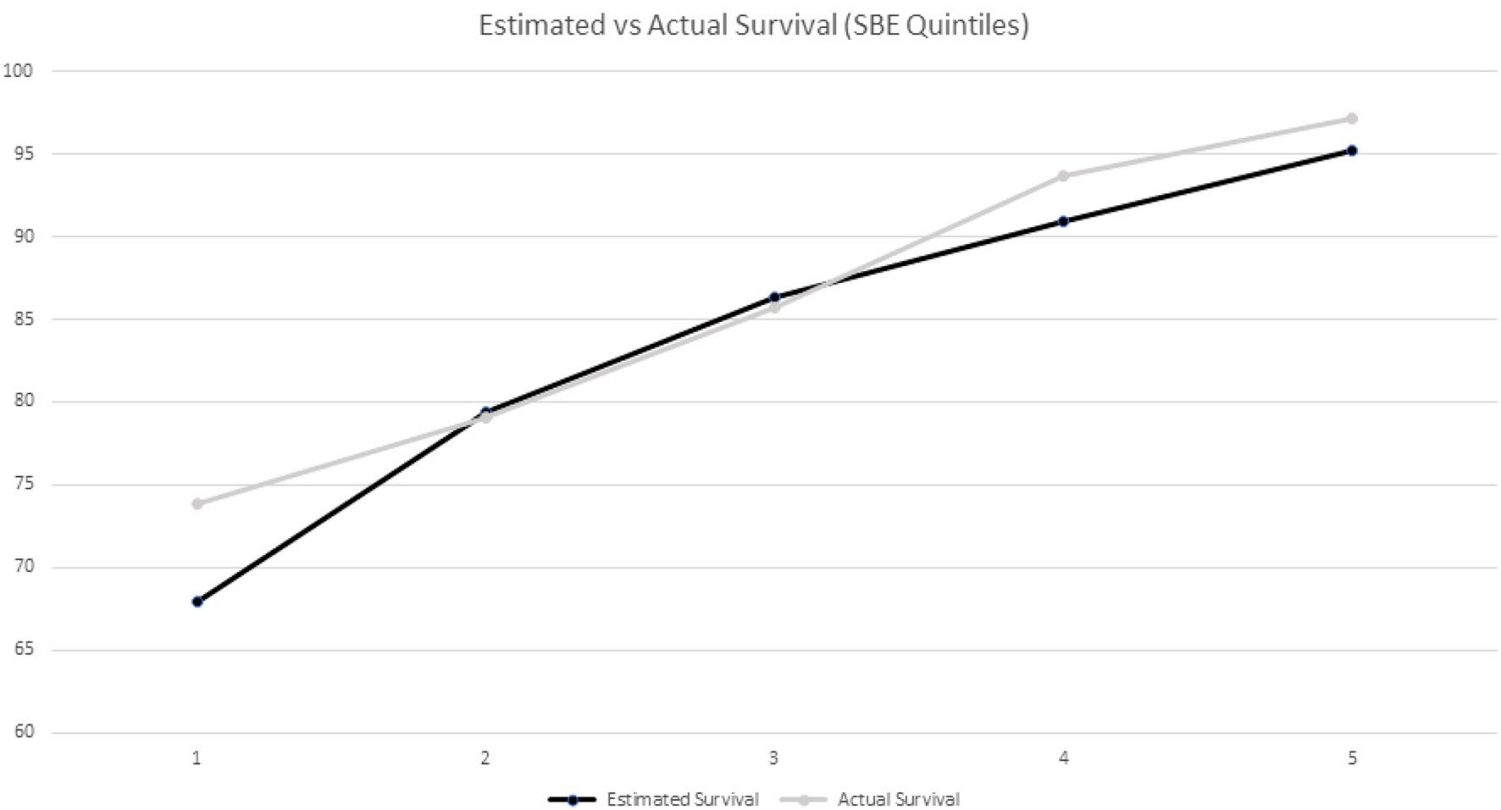
Comparison of post-transplant predicted vs actual survival according to SBE quintile distribution (1: worst; 5: best). X^2^(Q1 vs Q2): 1.416, P = 0.234; X^2^(Q2 vs Q3): 2.804, P = 0.094; X^2^(Q3 vs Q4): 6.606, P = 0.01; X^2^(Q4 vs Q5): 2.604, P = 0.107.

SBE was used to estimate 5-year patient survival at the time of transplantation. The study sample was divided into quintiles using the results of the SBE score. Figure 3 compares the average estimated survival rate with the actual survival in each SBE quintile.

Actual survival rates in our cohort were higher than predicted ones in the first and second highest and lowest SBE quintiles.

In unadjusted analysis, higher SBE score was a significant protective factor for recipient death [HR: 0.95 (0.93–0.96)], while higher KDPI and EPTS scores were significant risk factors for recipient death [KDPI, HR: 1.01 (1–1.02); EPTS, HR: 1.03 (1.02–1.03)]. All three scores were independently associated with post-transplant survival after adjustment (Table 3).

**Table 3:** Association between SBE, EPTS and KDPI scores and recipient death by Cox regression modeling.

	Unadjusted HR	95% CI	P value	Harrell’s C	AIC
SBE	0.945	0.931–0.959	< 0.001	0.694	− 2023.2
KDPI	1.011	1.004–1.018	0.003	0.570	− 1978
EPTS	1.026	1.020–1.032	< 0.001	0.710	− 2038.9

Model	Adjusted HR	95% CI	P value	Harrell’s C	AIC
Model 1^a^ SBE	0.944	0.929–0.959	< 0.001	0.718	− 2022.1
Model 2^a^ KDPI	1.010	1.003–1.017	0.006	0.646	− 1980.6
Model 3^a^ EPTS	1.027	1.020–1.033	< 0.001	0.735	− 2036

*AIC* akaike information criterion, *CI* confidence interval, *EPTS* estimate post-transplant survival, *HR* hazard ratio, *KDPI* kidney donor profile index, *SBE* survival benefit estimator.

^a^Models 1, 2 and 3 adjusted by recipient variables: gender, history of hypertension, ischemic heart disease, cardiac heart failure, peripheral vascular disease, stroke and hepatitis C infection.

Albeit patients with the worst SBE 5-year patient survival estimation (1st quintile) had a 23 median percentage point higher EPTS score and a 14 median percentage point higher KDPI score compared to the 2nd quintile group, actual survival between these two groups did not differ significantly (log Rank: 1.665; P = 0.197). This difference was not significant after adjustment for multiplicity.

Discrimination and calibration of SBE, EPTS and KDPI models are summarized in Table 3 and were tested with Harrell’s C statistic for Cox models. Discrimination was poor when using the KDPI model, with a Harrell’s C of 0.57. Using the SBE and EPTS scores improved discrimination when compared to the KDPI model, with a Harrell’s C of 0.69 and 0.71, respectively. Finally, adjusting also for clinically significant variables made a further improvement to a maximum c-statistic of 0.72 and 0.74 for the SBE and EPTS adjusted models, respectively.

## Discussion

Clinical tools aimed to optimize selection of donor-recipient pairs and estimate the potential survival benefit that a specific organ could involve for a specific recipient have been recently developed.

The present study evaluated the predictive performance of SBE in terms of 5-year patient survival after kidney transplant, even if the patient lost the graft during that time. In our sample, SBE, EPTS and KDPI score are associated with 5-year patient survival and they remained significant even after multi-adjusted survival analysis by recipient comorbidities (Table 3).

In our study SBE proved to be an independent post-transplant survival predictor both in unadjusted and adjusted analysis. SBE showed acceptable discriminatory performance, with a C-statistic of 0.69 that was close to that of EPTS (0.71) and higher than that of KDPI (0.57). Surprisingly, the calculated C-statistic for the SBE score in our sample was higher than that obtained in its development sample (0.69 vs 0.64)^12^. However, in this European population SBE tended to underestimate actual survival mainly in the subsets of patients with either highest or lowest SBE scores, therefore reflecting limited utility of this tool in these specific populations. Subjects within the worst and second-worst quintiles according to SBE estimated 5-year survival did not demonstrate significant differences in actual survival (Fig. 3). This is not an isolated finding; in a sample of KT performed in a Eurotransplant center Schulte et al. described a mortality rate of 16 percentage points higher in recipients with an EPTS score between 61–80, compared to those with an EPTS score > 80^27^. The absence of differences in survival between worst and second-worst quintiles in our sample could be due to higher prevalence of cardiovascular disease (i.e. congestive heart failure or peripheral vascular disease) in the second-worst quintile compared with the worst (Table 2), which is not considered when calculating the EPTS or SBE scores. Conceivably, donor and recipient risk factors may affect post-transplant survival differently in non-US population, with older age possibly being the predominant risk trait in our Spanish cohort. Additionally, the sample size of these two groups is underpowered to detect a difference of 5.2 percentage point in the mortality rates of the worst and second-worst quintiles.

EPTS score showed moderate discrimination in the original US data, with a C-statistic of 0.69^28^. Clayton et al*.* evaluated this score in 4983 kidney transplant recipients from the Australia and New Zealand Dialysis and Transplant (ANZDATA) Registry. The authors analyzed three different Cox models (EPTS only; EPTS plus donor age, hypertension status and HLA-DR mismatch; EPTS plus log-Kidney Donor Risk Index) and reported a Harrell’s C-statistic of 0.67, 0.68 and 0.69 for each model respectively^18^. We found similar discrimination power with a C-statistic of 0.71 in unadjusted analysis that improved to 0.74 when relevant clinical recipient variables were added to the model. EPTS was also tested as a prognostic tool after deceased-donor KT in Mexican patients, with an area under the receiver operating characteristic curve of 0.64^29^.

KDPI showed the lowest survival predictive power among the tools. This was an expected finding, as KDPI and Kidney Donor Risk Index were essentially designed as tools to estimate graft durability, but their potential application as predictors of post-transplant patient survival has been tested before. We found a 1.1% higher risk of mortality per percentage point of KDPI increase, and poor 5-year patient survival prediction capacity with a C-statistic of 0.57 in unadjusted analysis that improved to 0.65 when additional recipient variables were added to the analysis. Similar results were obtained by Peters-Sengers et al., reporting 5-year mortality C-statistic for Kidney Donor Risk Index (including deaths after graft loss) of 0.68^30^. Calvillo-Arbizu et al. also suggested that KDPI could constitute a potential indicator of patient survival, especially for recipients older than 60 years^31^. However, in another study with Spanish transplant patients no relationship was found between KDPI score and recipient death^17^.

The observed disparities between our results and American studies could be explained by several relevant demographic differences between the US and Spain. For instance, more than 90% of our donor and recipient sample was constituted by Caucasians whereas the cohort used in the development of SBE was much more ethnically diverse^12^. In addition, Spanish recipients were older but with less prevalence of diabetes, shorter dialysis vintage and lower sensitization degree. Cold ischemia time was also shorter in our sample. These differences were translated into a lower EPTS score compared to that of Bae’s study. On the other hand, our donors were older and more frequently diabetics and with hypertension, which translated in a higher KDPI compared to that registered in SBE development sample^12^.

Divergences in transplant era can also be a possible explanation of score performance discrepancies, as pointed by Rose et al.^32^. The Cox proportional hazards regression model used to develop Kidney Donor Risk Index was obtained from a sample of US kidney recipients from 1995–2005. Donor and recipient characteristics have changed significantly over the last 20 years, both in the US and in Europe^33,34^, as well as other factors such as dialysis-associated mortality or waitlist removal^35^. In addition, the increasing prevalence of chronic kidney disease, the growing demand for organ donations and the incorporation of expanded criteria donors as a viable organ source have modified even further the current transplant landscape in the last decades.

Differences also affect transplant policies and organ allocation. Between 2004 and 2015 more than 50% of the retrieved kidneys with KDPI > 85% were discarded in the US, even after the initiation of the KDPI era^36,37^. Although in Spain there are no official data regarding KDPI-associated discard rates as the use of KDPI is not routinely extended, Arias-Cabrales et al. reported that 35.5% of patients in their study received a kidney from a KDPI > 85 donor^17^, a percentage which was slightly lower (30.6%) in our current sample.

Results may also depend on other donor, recipient and procedure characteristics not accounted for during calculation of SBE, KDPI or EPTS scores, such as graft damage or abnormalities, excessive first warm ischemia time, additional recipient comorbidities or likelihood of transmission of diseases during transplantation. Additionally, divergences in health care systems between the US and Spain could explain some of the observed discrepancies. More than 70% of US kidney transplant patients report economic problems to access immunosuppressive medication^38^, with Medicare coverage for those drugs being lost after the first three years after transplantation^39^. In comparison, the transplant procedure, follow-up monitoring and all associated medication is completely covered in Spain and other European countries^40^, regardless of transplant duration.

The present analysis has some limitations. Extensive efforts were undertaken to adjust for potential confounding, but residual confounding due to unmeasured variables is still possible. Moreover, most of our sample consisted of Caucasians. Therefore, our results cannot be extrapolated to subjects of other ethnic groups. Likewise, most of the donors in this study were brain-dead so the studied scores may perform differently in other settings such as donation after cardiac death. Additionally, we only tested the predictive capabilities of the SBE score at the 5-year time point. As a consequence, our results should not be extrapolated to other time points. Finally, organ allocation strategies and policies may diverge considerably between countries and, to a lesser extent, between transplant centers, which may affect the predictive potential of scores such as SBE.

But the present study has several strengths too. To our knowledge, this is the first study that offers an external validation analysis of the SBE score in European population. SBE scoring considers both donor and recipient characteristics to build a post-transplant survival estimation. Although the discrimination power was similar to that of EPTS in our sample, due to recipient traits being the most relevant to determine recipient survival, the inclusion of donor associated variables provides a small but important improvement to survival prediction, thanks to their significant effect on future graft function. Our sample was built using data from two different transplant centers and included survival information regardless of actual graft function.

In sum, this is one of the first studies to provide external validation of the use of the SBE score as a standalone score in a non-US sample of kidney transplant recipients. Further analysis should be performed to adequately characterize its potential to produce accurate and individualized post-transplant survival predictions.

## Data Availability

The datasets generated during and/or analyzed during the current study are available from the corresponding author on reasonable request.

## Abbreviations

AIC: Akaike information criterion
ANZDATA: Australia and New Zealand Dialysis and Transplant Registry
CI: Confidence interval
CIT: Cold ischemia time
DCD: Donation after cardiac death
Dis: Disease
DV: Dialysis vintage
EPTS: Estimated post-transplant survival
HR: Hazard ratio
HTN: Hypertension
IQR: Interquartilic range
KAS: Kidney allocation system
KDPI: Kidney donor profile index
KT: Kidney transplantation
OPTN: Organ Procurement and Transplantation Network
PRA: Panel reactive antibodies
Q: Quintile
SBE: Survival benefit estimator
SCr: Serum creatinine
SD: Standard deviation
